# Novel multi-mode shortwave broadcast transmitting antenna array

**DOI:** 10.1038/s41598-022-15336-x

**Published:** 2022-06-30

**Authors:** Wang Yao, Huotao Gao, Ying Tian, Taoming Lu, Xiaolin Zhang

**Affiliations:** 1 School of Electronic Information, Wuhan University, Wuhan, 430072 China; 2 38th Research Institute of China Electronics Technology Group Corporation, Hefei, 230088 China

**Keywords:** Electrical and electronic engineering, Techniques and instrumentation

## Abstract

Currently, shortwave broadcasting in the range of 5.9–26.1 MHz remains a relatively large blind spot within 900 km owing to the limitations of ionospheric characteristics. Reducing the emission frequency is a feasible approach for covering blind spots and improving broadcast performance. Thus, a new type of shortwave broadcasting antenna array capable of reducing the lowest emission frequency to 4.4 MHz is proposed in this paper. An electromagnetic simulation software is used to optimize the design. The simulation analysis shows that for the 4 × 4 multi-mode shortwave broadband transmitting antenna array, the gain obtained is 12–23.5 dB in the 4.4–27.4 MHz frequency band, and the VSWR for each mode is lower than 2.5. The radiation patterns at 5.9 MHz and 4.4 MHz on a vertical plane are compared, and the results prove that the radiation elevation angle of the new transmitting array increases significantly. The larger elevation angle and lower frequency ensure the enhancement of close-range coverage. A scale model prototype is fabricated and characterized, and the results of the measurement agree well with those of the simulations. It provides a theoretical basis and technical support for the improved design of broadband high-power shortwave broadcasting transmitting antenna systems.

## Introduction

The high-power shortwave (SW) antenna system can realize long-distance and even intercontinental communication through surface wave/sky wave propagation. These systems are built inland and require the ionospheric refraction of radio waves several hundred kilometers above the Earth’s surface to overcome the line-of-sight limitation caused by the Earth’s curvature^1,2^. Surface wave antenna systems face difficulties in pushing the range limit of 400 km owing to various reasons such as high attenuation and vertical incidence ionospheric clutter problems^3,4^. Therefore, sky wave antenna systems show significant potential for global broadcasting. The large detection range of sky wave propagation (up to 3500–4000 km) has garnered a considerable amount of attention from global institutes^5,6^.

As a signal transmitter, SW broadcast antennas must meet certain key requirements, including a wide bandwidth (more than one octave), high gain (above 20 dB), and multiple transmitting modes with various horizontal/vertical radiation patterns. A simple and reliable mechanical structure can withstand the erosion from wind and rain^7,8^. Curtain antennas are a typical form of SW high-power transmitting antennas, consisting of a dipole array, mesh reflector, and assorted fixed installation^9–15^. The first 500 kW SW broadcasting antenna, designed by Stephen W. Kershner in 1968^9^, had dual-row cage antennas set as the radiation elements and reached 1.5 octaves. Cave antennas can provide a wide bandwidth and have sophisticated constructs; however they suffer from large dimensions. In 1988, Martin et al. proposed a curtain antenna to simplify and miniaturize the elements by combining the features of cage antennas and folded dipoles^10^. The folded dipole can halve the length of the dipole and fit a four-column array of the same width. Thomson-CSF Company, France, invented 360° rotatable SW antennas with dipoles in the 1990s^16,17^, and these could flexibly control the horizontal radiation patterns. However, rotation results in new challenges in the operation of the antenna, a lighter weight, and a more stable structure. Thomson-CSF, formerly known as Thales Group, subsequently launched a series of rotatable antennas to achieve an improved performance and increase the maximum detection range to 8000 km^18^. The maximum bandwidth of a single array exceeded one octave (5.9 MHz–12.17 MHz).

**Figure 1:**
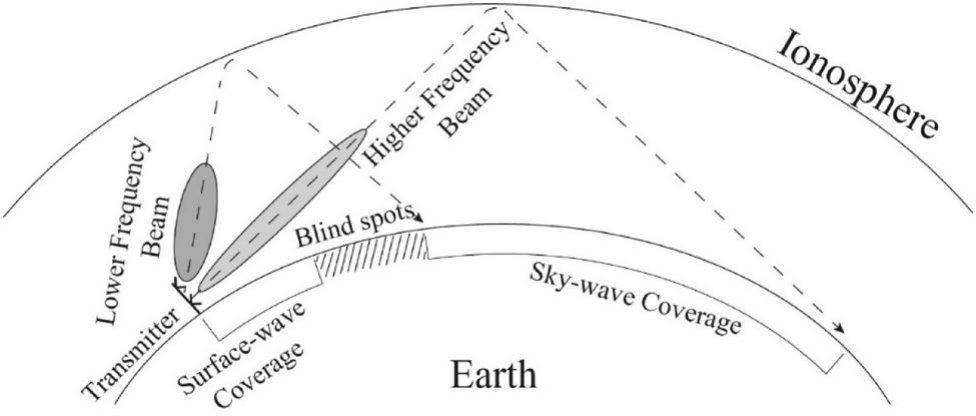
Illustration of sky-wave propagation.

The long-range coverage capability can meet the current demand; however, the problem of insufficient short-range coverage capability has emerged: the blind spot during SW broadcasting is between the maximum range of ground wave propagation and the minimum range of sky wave propagation. Figure 1 shows a blind spot in a sky wave propagation system. The minimum coverage distance (one skip) of SW broadcasting is limited by the ionosphere position and main beam angle, and it cannot reach the target within 500 nmi (approximately 926 km)^19^. Nearly 500 km of blind spots exist between the surface wave and sky wave propagations. Owing to the conductivity of the earth, the elevation angle of the antenna's main beam decreases as the frequency increases. A greater elevation angle gives rise to the potential for closer detection distances. Owing to certain properties of sky wave propagation, increasing the elevation angle overall by using a phase shifter or by pitching up the wave front is not useful. Higher frequency rays can escape through the ionosphere with a large elevation angle and low plasma density^20^.

Near vertical incidence sky wave (NVIS) propagation allows SW ionospheric communication over relatively short distances, typically up to 400–500 km. The operation frequencies are usually defined between 2 and 5 MHz at nighttime and between 5 and 10 MHz at daytime^21–26^. Therefore, expanding the spectrum to a lower frequency (below 5 MHz) presents an effective solution to compensate for short-range detection and ensure the existing coverage. Owing to cost constraints and limited mechanical loads, solely increasing the electric dimensions of dipoles is not optimal for rotatable SW antennas. Miniaturization and lower frequencies constitute appropriate methods of complementing the coverage of the SW antenna, which must be solved urgently.

**Figure 2:**
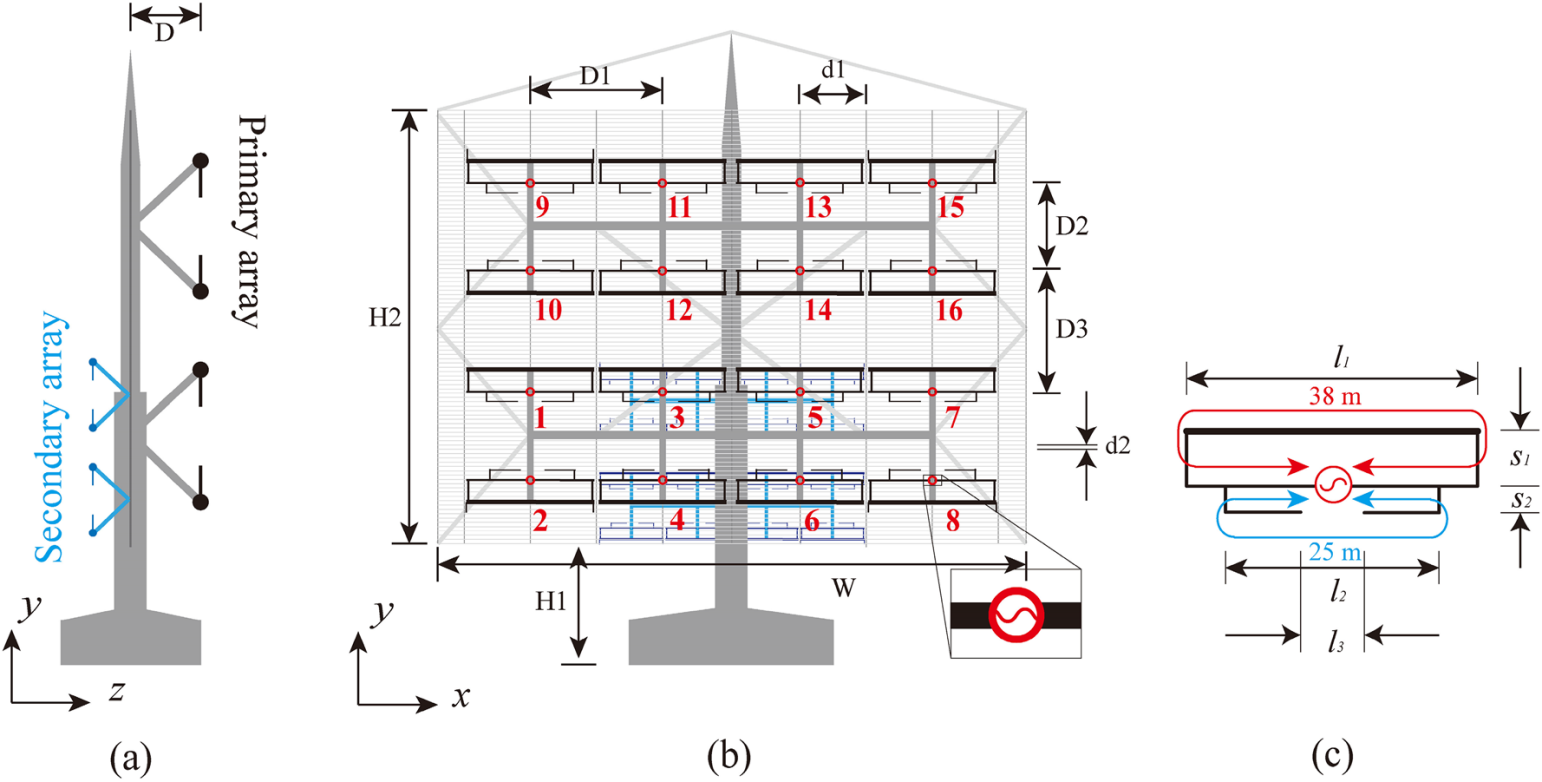
Antenna for SW broadcasting: (**a**) side view, (**b**) front view, (**c**) modified folded dipole. D = 9 m, D1 = 18 m, D2 = 15 m, D3 = 19 m, d1 = 9 m, d2 = 0.7 m, H1 = 15 m, H2 = 63 m, W = 75 m, *l*_1_ = 17 m, *l*_2_ = 11.5 m, *l*_3_ = 4 m, *S*_1_ = 2 m, and *S*_2_ = 1 m.

In this study, we demonstrate a SW broadcast antenna array that operates in the 4.4–13.7 MHz (the primary array) and 8.8–27.4 MHz (the secondary array) ranges (as shown in Fig. 2). The primary array consists of a 4 × 4 array, mesh reflector, and supporting swinging strut. The secondary array was a 1:2 scaled prototype of the primary array. Each element is a modified folded dipole composed of a main folded dipole and additional branches. The impedance in the 3rd mode can be appropriately increased by introducing a smaller branch loop (which is precisely in the antenna mode at this frequency). After the wire diameter is optimized, the modified folded dipoles can exhibit an octave impedance matching (7–13.7 MHz). Simultaneously, a mirror surface current is induced in the adjacent element using mutual coupling, which can significantly reduce the impedance of each element in the first mode (transmission mode). The minimum matching frequency of each element can be reduced to 4.4 MHz without extending the electrical dimensions. The total dimension is equivalent to that of the product from Thales Company, which is a competitor, and the lower frequency band is expanded by 0.65 octave.

To cover a closer range, a smaller incident angle for the radio waves is required. Lowering the frequency implies lowering the critical angle of total reflection, which significantly enhances the coverage of a closer target within 900 km, filling the blind spot between the sky wave and surface wave coverage. The HF broadcast antenna can be placed on a 360° horizontal rotatable mechanical platform. In the following sections, we describe in detail the radiation characteristics of the HF broadcast antenna. A 1:200 scale model prototype was fabricated and used for measurements to verify the advancement and feasibility of this scheme.

## Design and characteristics

### Modified wideband folded dipole

Figure 2c depicts the schematic diagram of the proposed modified wideband folded dipole composed of two dipoles at different frequencies: the longer one 38 m in length (equal to the half-wavelength at 4 MHz) and the 25 m shorter one (equal to the half-wavelength at 6 MHz). The folded dipole could halve the length of the dipole and achieve a broader bandwidth, while having a characteristic impedance of approximately 600 Ω.

**Figure 3:**
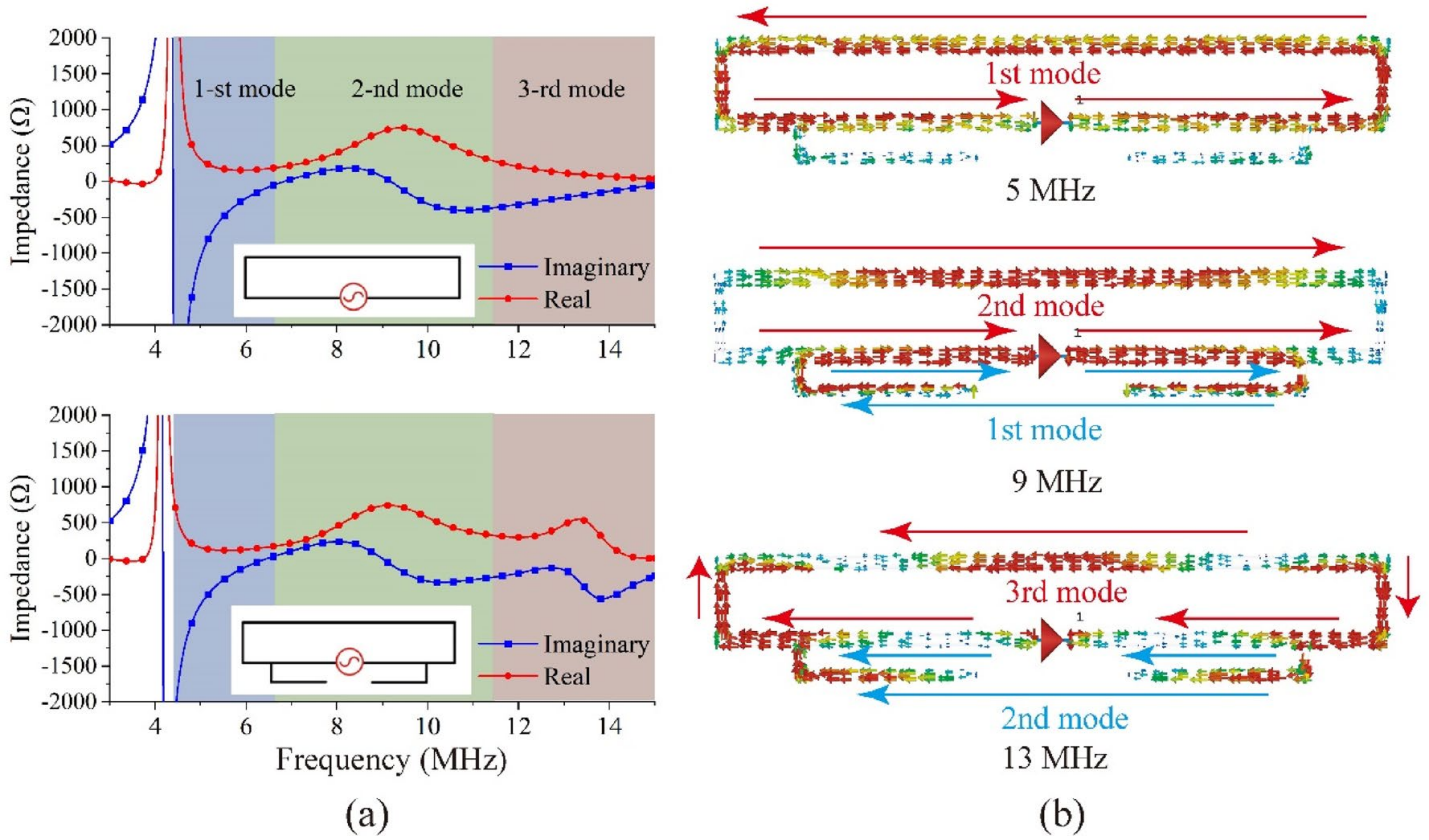
(**a**) Impedances of the initial folded dipole and the modified folded dipole, (**b**) surface current of modified folded dipole in three modes.

The impedance and surface currents for the single folded dipole were obtained by simulation using CST Microwave Studio. Figure 3a shows the real and imaginary parts of the impedance. The transmission line mode (1-st mode) of the initial folded dipole began at 4.4 MHz and the folded dipole was equal to a half-wavelength dipole. In addition, there were two higher-order resonant modes: the antenna mode (2-nd mode) began at 6.8 MHz and the third mode began at 11.5 MHz. The simulated impedance illustrated in Fig. 3a indicated that the antenna mode could provide good matching characteristics (the characteristic impedance of the dipole was 600 Ω, which significantly deviated from the real parts of the first and third modes).

**Figure 4:**
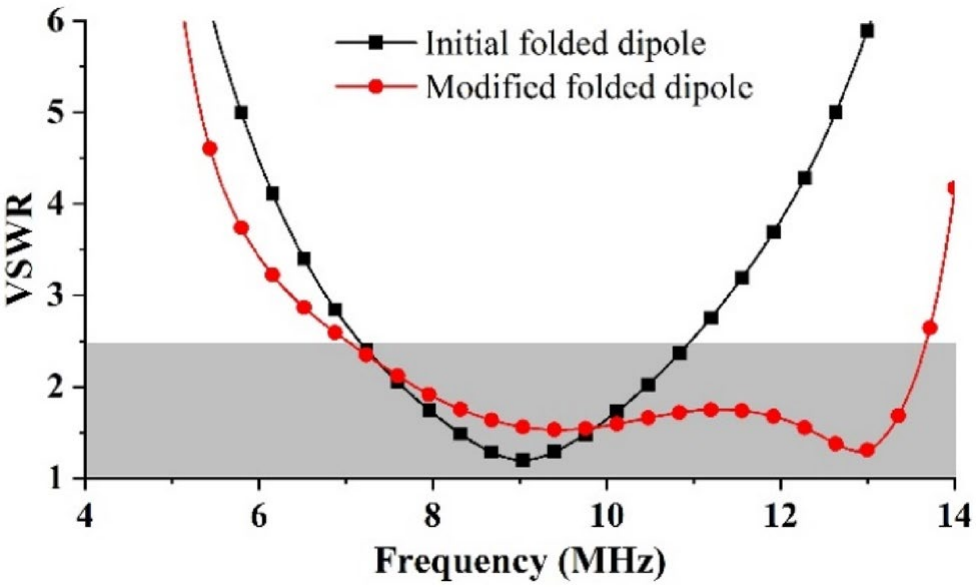
Simulated VSWRs of initial and modified folded dipole.

The modified folded dipole with the higher-frequency resonant branch could achieve good impedance matching in the third mode, which benefitted from the branch active in the antenna mode. The surface currents of the modified folded dipole in the three modes are shown in Fig. 3b; when the main dipole was in the second and third modes, the branch was in the first and second modes, respectively. The simulated results of the impedance also verified this phenomenon, and the real part increased in the third mode. The VSWR results were calculated by CST MWS, including the initial dipole and the modified dipole. The initial folded dipole exhibited a narrow bandwidth in the second mode. By introducing a higher-frequency resonant branch, the VSWR in the third mode was significantly reduced to below 2.5 in the range of 6.8 to 13.7 MHz, and the simulation result is shown in Fig. 4. However, the impedance matching was remained poor in the first mode, and it showed significant volatility.

### Mutual coupling and elements’ optimization

Mutual coupling occurred among the elements, which altered the performance of the array antenna. The rotatable antenna needed to reduce the array spacing to the largest extent, which rendered mutual coupling inevitable. In this design, therefore, the interaction and mutual effects amongst the elements must be considered. The input impedance of the antenna in the presence of other elements or obstacles, which was treated as the driving point impedance, depended on the self-impedance (input impedance in the absence of any obstacles or other elements) and the mutual impedance between the driven element and other obstacles or elements.

**Figure 5:**
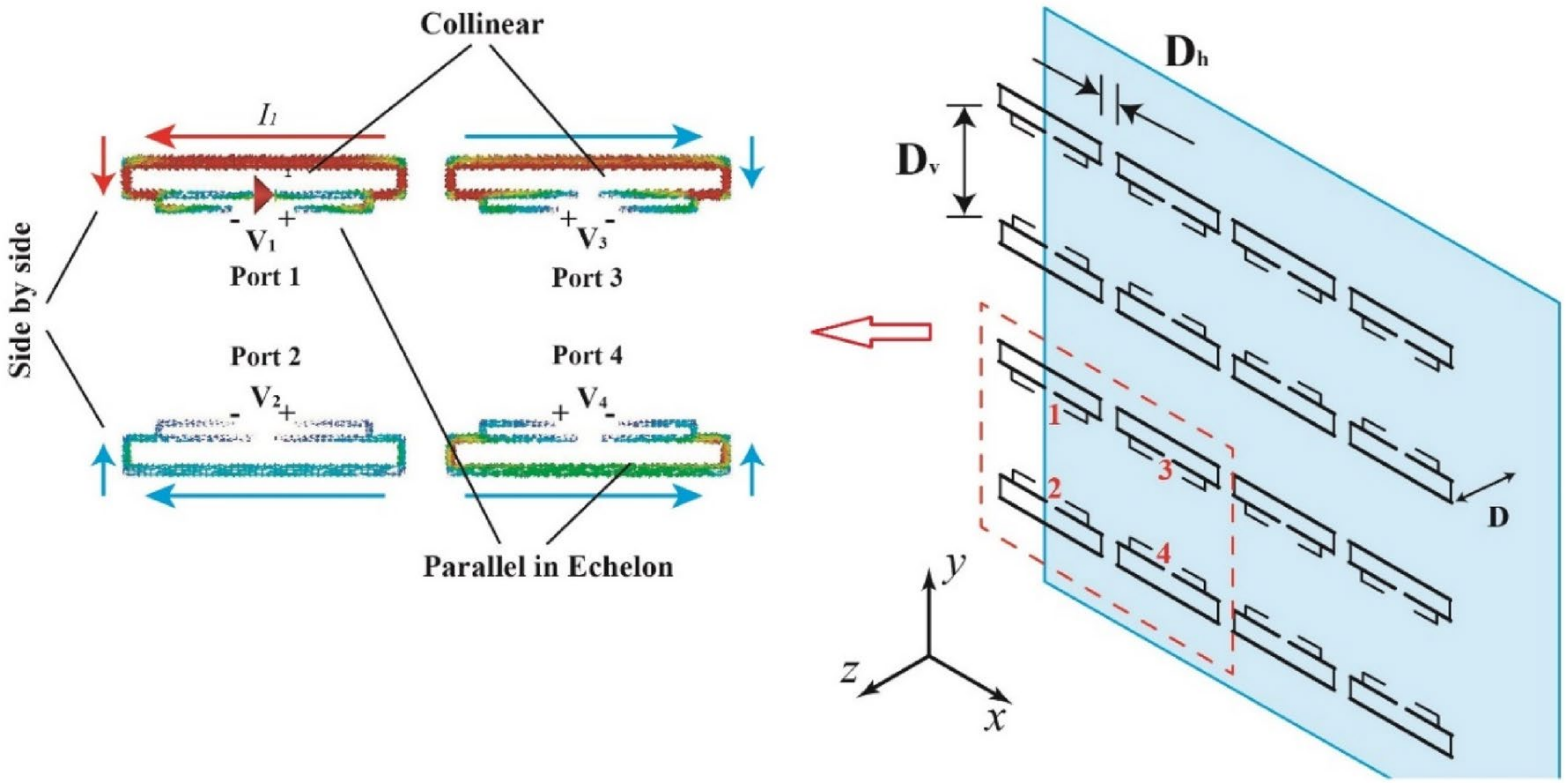
Mutual coupling in the sub-array.

**Figure 6:**
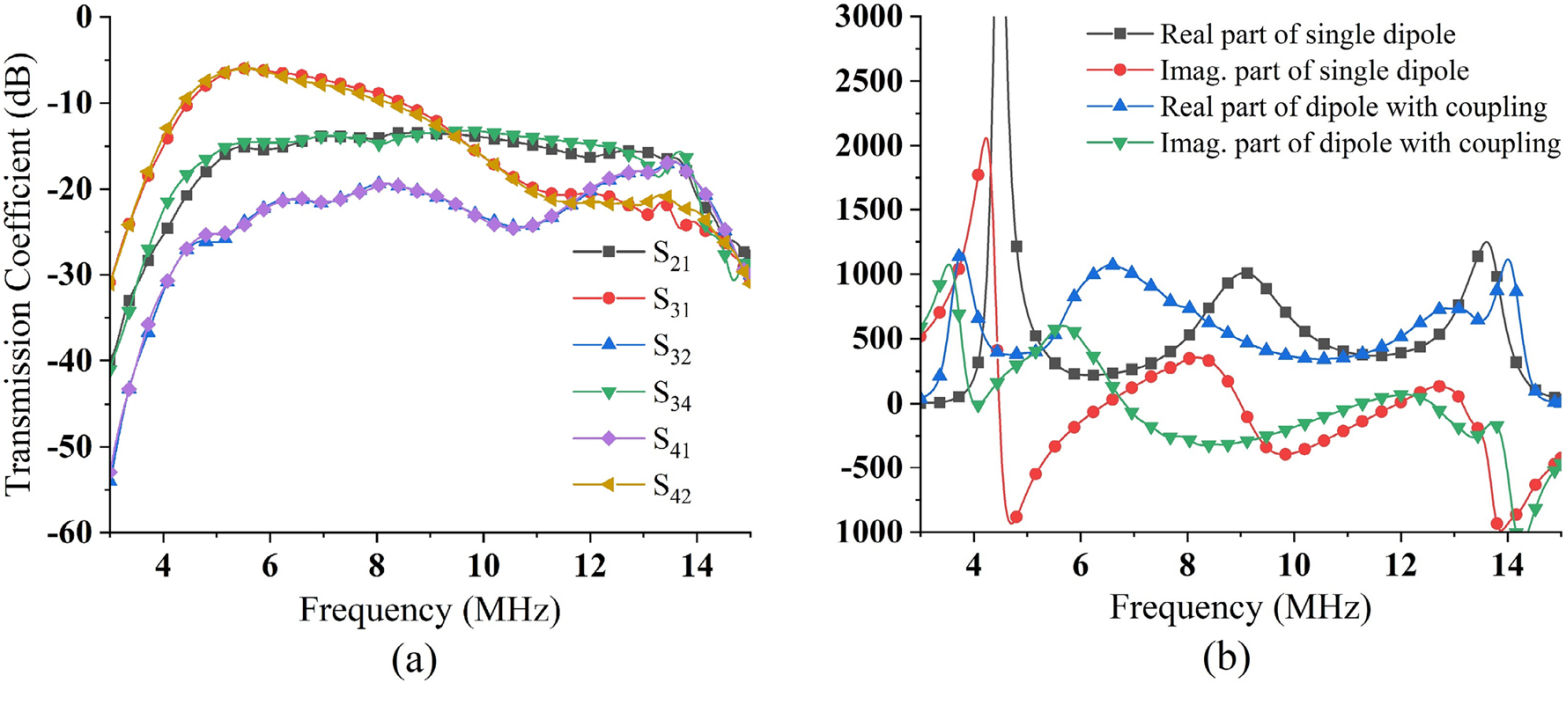
(**a**) Transmission coefficient between four ports (D_h_ = 1 m), (**b**) impedances of the dipole before and after coupling.

This program has planned to adopt a 4 × 4 unit array to achieve HF broadcasting. To briefly analyze the mutual coupling phenomenon, the array could be processed into four sub-arrays arranged in a 2 × 2 array for analysis, and each sub-array is shown in Fig. 5. For a two-element array of linear dipoles, there existed three classic configurations for mutual coupling called side-by-side, collinear, and parallel-in-echelon, which are shown in Fig. 6. In this figure, port 1 is the sole excited port, and the remaining three are open circuited. *Z*_12_ = *Z*_21_, *Z*_13_ = *Z*_31_, and *Z*_14_ = *Z*_41_ for a reciprocal network; therefore, the driving-point impedances could be expressed as

1 $$ \begin{aligned} Z_{1d} & = Z_{11} + Z_{12} + Z_{13} + Z_{14} = Z_{11} + Z_{21} + Z_{31} + Z_{41} \\ & \left. {= \frac{{V_{1} + V_{2} + V_{3} + V_{3} }}{{I_{1} }}} \right|_{{I_{2} ,I_{3} ,I_{4} = 0}} \\ \end{aligned} $$

The surface current of the sub-array simulated using CST MWS is shown in Fig. 5. For the side-by-side section, the excitation voltage V_1_ and the voltage V_2_ at port 2 have the same sign, which would increase the impedance in port 1. Thus, the increased longitudinal spacing in the antenna array could decrease the mutual coupling in the side-by-side section. Satisfactory phenomena appeared in the collinear and parallel-in-echelon sections, and the signs of the voltages V_3_ and V_4_ were opposite to that of the excitation voltage V_1_. By strengthening these two parts with reduced lateral spacing, the drive-point impedance could be significantly suppressed.

The HF antenna was simplified to further investigate the mutual coupling in the full array, as shown in Fig. 5. The 4 × 4 array was placed in front of a PEC wall at a distance of D = 9 m (a quarter of the wavelength at the center frequency). The longitudinal spacing D_v_ was set to 15 m to prevent mutual coupling in the side-by-side section. However, owing to the collinear and parallel-in-the-echelon sections, the lateral spacing D_h_ was set to be much smaller than D_y_, which and ensured the compactness of the array. According to the symmetry of the array, the four ports marked in Fig. 5 represented all the ports. The transmission coefficient simulated by CST MW studio shows that the coupling of collinear section is significant in the frequency range of 4–8 MHz (S_31_ and S_42_ are close to 0.5). This coupling helps to reduce the impedance of the dipole, as shown in Fig. 6b, the impedance of the first mode of the dipole is reduced from several thousand ohms to 1000 ohms, enabling better impedance matching.

**Figure 7:**
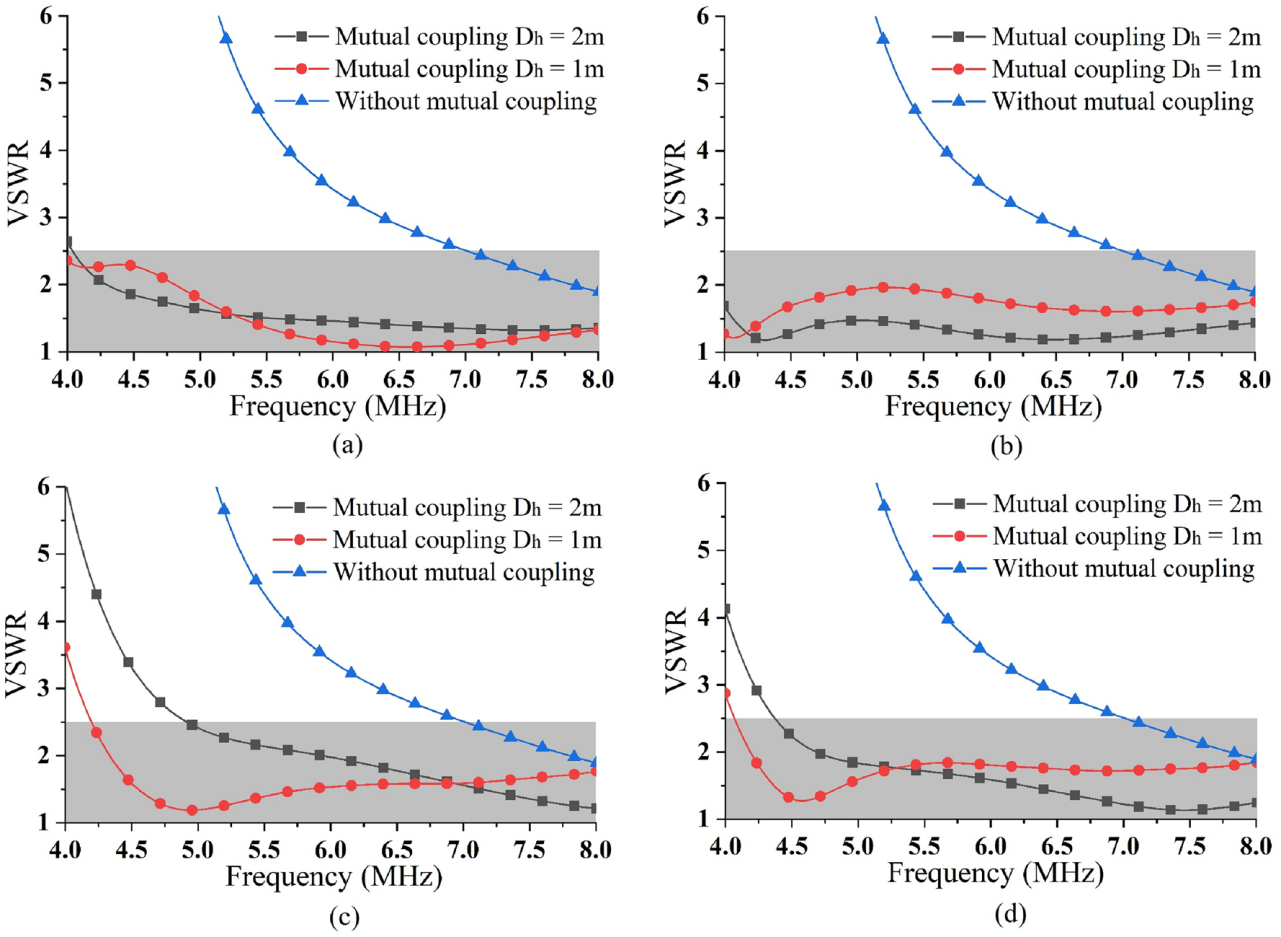
Simulated results of VSWR for (**a**) Port 1, (**b**) Port 2, (**c**) Port 3 and (**d**) Port 4.

The simulation results of the VSWRs of the four ports also corroborated this conclusion, as shown in Fig. 7 (which listed the VSWRs of the four ports in the case of two lateral spacings). As the D_h_ decreases, the collinear section coupling increases, with better matching around 4 MHz. A certain safety distance needs to be maintained between the two elements (to avoid breakdown discharge or short circuit caused by an unexpected swing), and the D_h_ less than 1 m will not be considered. The VSWR of each port was significantly lower than that of a single element, which facilitated the realization of a better result at a lateral spacing of 1 m. The VSWR was less than 2.5, the frequency was as low as 4.3 MHz, reached the preset target.

### Feeding network, reflection curtain and metal support

Figure 2 depicts the overall structure of the rotatable HF antenna composed of two antenna arrays (the primary array for 4.4–13.7 MHz and the secondary array for 8.8–27.4 MHz), a reflection curtain, and a steel support structure. The parameters of the primary array were the same as those mentioned above, and the secondary array was a 1:2 scale model prototype of the main array. It should be emphasized that the two adjacent rows of elements were placed symmetrically, and the metal arm was fixed onto the thicker wire of the antenna. Owing to the element being symmetrical, the zero potential point was located at the midpoint of the wire, and thus the fixation at this point had affected the antenna the least. The horizontal distance for each port was D1 = 18 m, the vertical distance for rows 1 and 2 (D2) was 15 m, and that for rows 2 and 3 (D2) was 19 m. This arrangement could ensure that the phase centers of each row of elements were equally spaced, and the side lobe of the radiation pattern could be reduced simultaneously.

The reflector of the antenna was H1 = 15 m above the ground, and the height and width of the reflection curtain were H2 = 63 m and W = 75 m, respectively. Owing to the large overall size of the antenna, the flat reflector must withstand strong wind resistance; therefore, the rotatable antenna typically uses a metal mesh as a reflector. To ensure the efficiency of reflection and the front-to-back ratio of the antenna, the pitch of the mesh was less than one-tenth of the wavelength corresponding to the highest frequency. The antenna was horizontally polarized; therefore, it was sufficient to ensure that the horizontal grid spacing d2 met the wavelength requirements (d2 = 0.7 m, less than 1/10 of the wavelength at 26 MHz). The vertical grid acted as a fixed horizontal grid with a spacing of d1 = 9 m.

**Figure 8:**
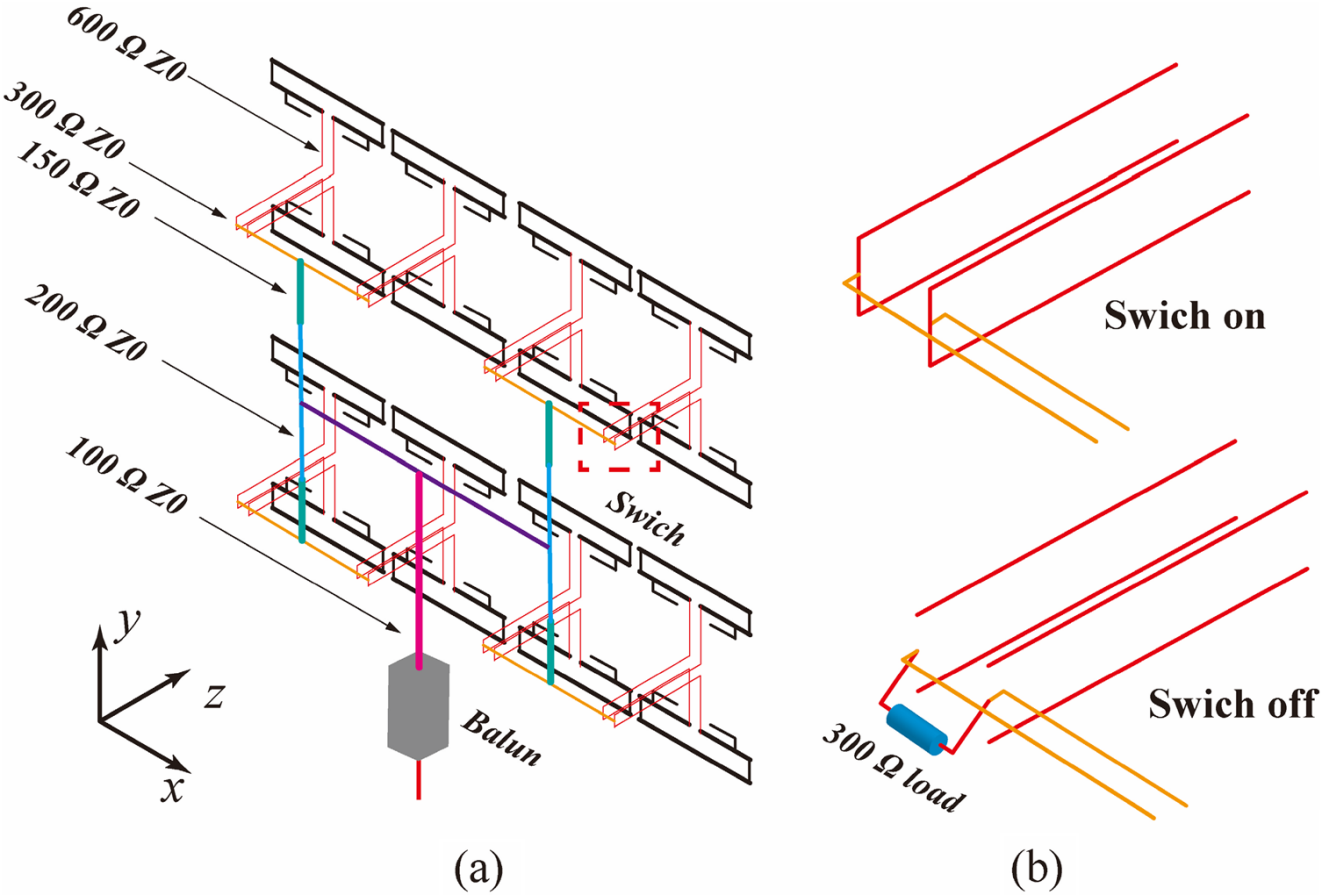
(**a**) Feeding network of the antenna array, (**b**) switch.

The antenna array was fed by a balanced transmission line with a diameter of 1–11 cm, and different impedances were achieved by adjusting the spacing between the two lines. The overall feeding network is shown in Fig. 8, and the dimensions of various transmission lines are summarized in Table 1. The first-level transmission line (marked in red) is connected to two adjacent elements and subsequently connected to the second-level transmission line (marked in yellow) through a configuration switch. The switch can be simplified as a double-pole double-throw switch. When the radiation elements controlled by it needs to be turned on, its state is ON, as shown in Fig. 8b. To turn off, a 300 Ω load is connected to maintain the impedance matching of the system. The high-order transmission line was then merged to a balanced port with an impedance of 100 Ω. The different modes of transmission could be switched by controlling the configuration switches. Finally, a parallel resonant cavity balun with a 1:4 impedance transformation is usually used to convert the main feeder to coaxial^27^.

**Table 1:** Dimensions of balanced transmission lines.

Characteristic impedance	600 Ω	300 Ω	200 Ω	150 Ω	100 Ω
Line diameter (cm)	1	1	5	9.4	11
Line spacing (cm)	100	15.6	15.6	15.6	15.6

## Results

An antenna array with the reflector and support, as shown in Fig. 2, was modeled using CST MWS, and the boundaries in five directions of it, except the − y direction, were set as open boundaries. The HF broadcast antenna is usually located at the seaside; therefore, it covers the ocean, achieving transoceanic coverage. The boundary in the − y direction was set to be a conducting wall with a conductivity of 3.53 S/m (when seawater was at 25 ℃ with a salinity of 15 ppt)^28,29^. Using different excitation methods, the antenna array could obtain four operating modes, which are referred to as HR 2/2, HR 4/2, HR 2/4, and HR 4/4^30,31^. The array selected the appropriate mode according to different targets because the radiation characteristics of each mode were different.

### Simulation results of each mode

**Figure 9:**
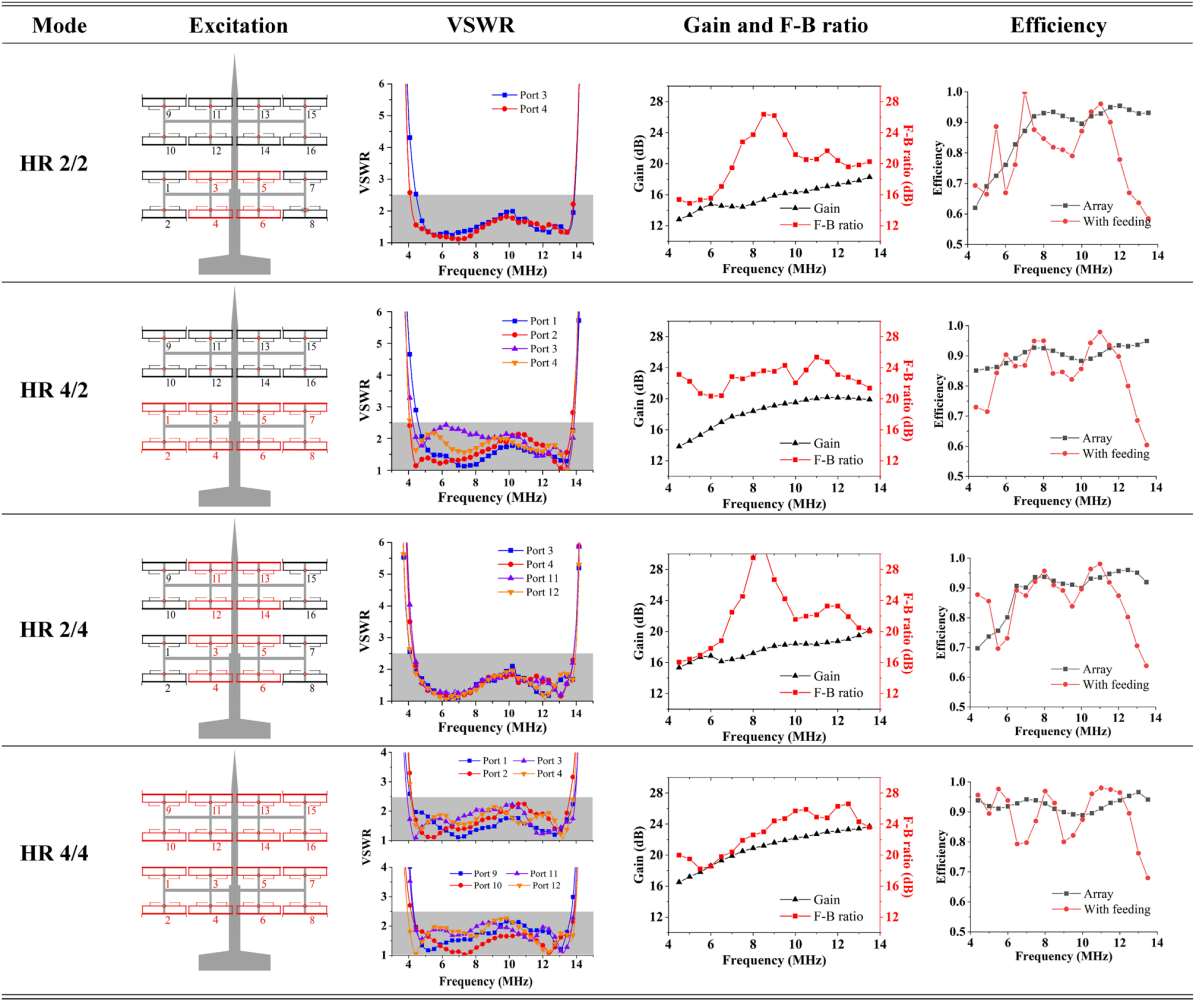
Characteristics of each mode.

**Figure 10:**
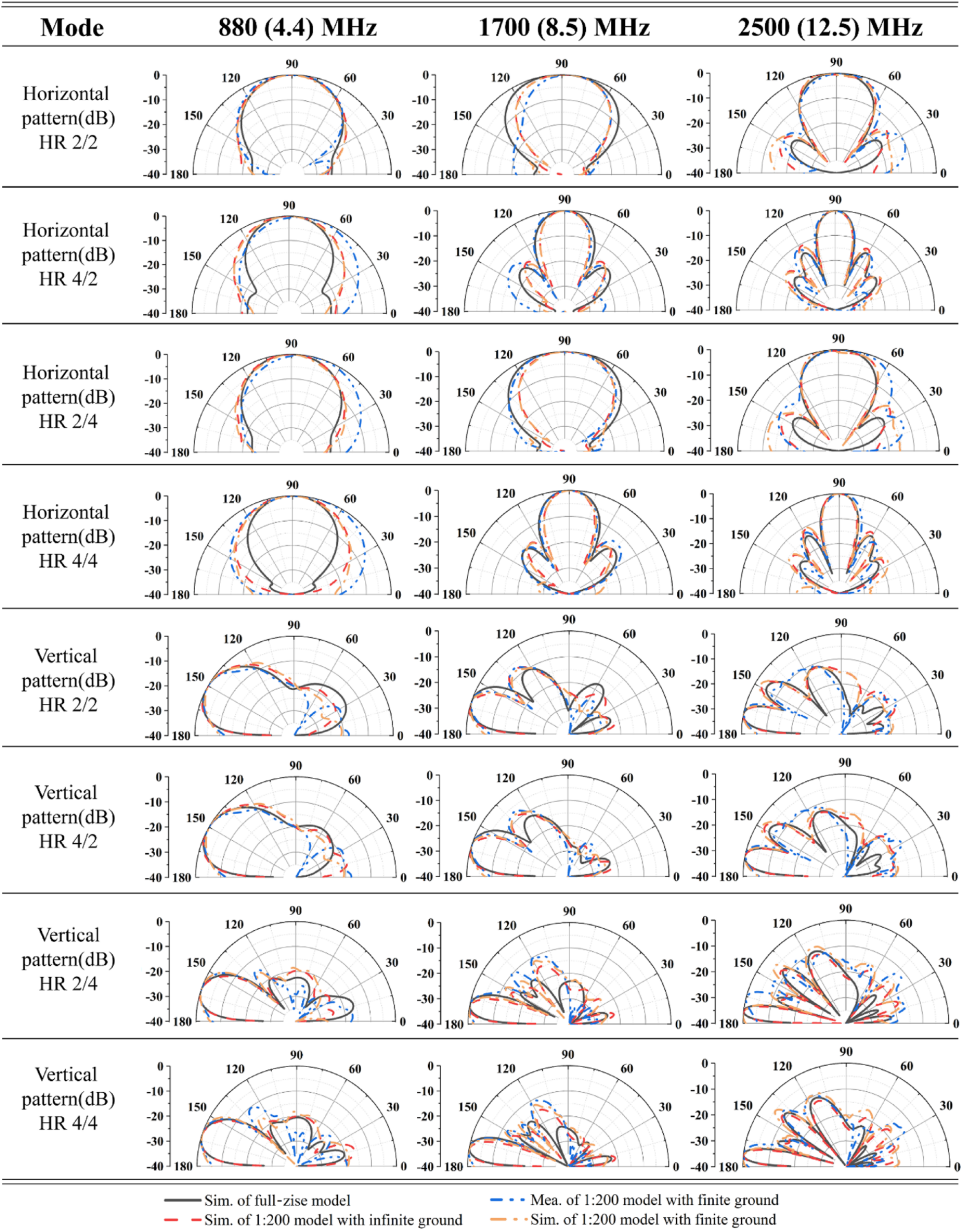
Simulation and measurement of patterns for each mode.

Figures 9 and 10 presents the specific simulation parameters of the antenna array in the four modes: excitation form, VSWR, gain and front-to-back ratio, and horizontal/vertical plane patterns. The VSWR of each port in the 4.4 to 13.7 MHZ frequency band was lower than 2.5 in each mode, owing to the symmetry of the ports, and the left ports are listed in the table. All ports were excited by equal amplitudes and were in phase. Owing to the conducting wall boundary condition along the − y direction, the main lobe of the radiation pattern would have an elevation angle that decreased as the operating frequency increased. Strictly speaking, the backscattering direction of the proposed antenna points into the ground, so the backscattering value in this paper takes the maximum gain in the range of 0–90° (vertical pattern in Fig. 10).

In the HR 2/2 mode, the radiation pattern of the array had a wide beam width (both in the horizontal and vertical planes) and a large elevation angle. The horizontal simulation results showed that the antenna had a wide lobe in the complete frequency band, and the horizontal − 3 dB beam width varied from 48.7° to 29.8° in the frequency band. The main lobe had a maximum elevation angle of 28° at the lowest frequency, which decreased to 10° as the frequency increased. In the operating frequency band, the gain exceeded 12 dB and was less than 18 dB, and the front-to-back ratio exceeded 15 dB. In this mode, the antenna array obtained a wider beam with a larger elevation angle, which was suitable for close and wide-range broadcast transmissions.

The HR 4/2 mode added the bottom four elements on the basis of the HR 2/2 mode while keeping the vertical beam width and elevation angle constant. The gain increased when the horizontal beam width was narrowed. The vertical plane pattern remained consistent with the elevation angle and beam width of the HR 2/2 mode. The horizontal − 3 dB beam width was narrowed down to 41.6°–16.5°, and the gain was increased to 14–20 dB. In this mode, the front-to-back ratio was greater than 20 dB. The elevation angle remained constant and the gain increased; therefore, it was suitable for regional broadcast transmissions in areas with close distances.

The HR 2/4 mode added four elements above the longitudinal direction of the HR 2/2 mode, which increased the gain while ensuring that the horizontal beam width remained constant. The horizontal beam width of the pattern remained identical to that of the HR 2/2 mode. However, owing to the increase in the vertical elements, the vertical beam width was narrowed, the gain was improved to 15–20 dB, and the front-to-back ratio exceeded 16 dB. The maximum and minimum elevation angles in the vertical plane were reduced to 18° and 6°, respectively. A large horizontal beam width, high gain, and small elevation angle made this mode suitable for long-distance and wide-range broadcast transmission.

In the HR 4/4 mode, the 16 elements of the array were completely open, and the antenna achieved maximum gain. The elevation angle of this mode was consistent with that of the HR 2/4 mode, and the − 3 dB beam width of the main lobe was narrowed down to 42.3°–16.7° in the horizontal plane and to 19.6°–6.5° in the vertical plane. Additionally, the gain increased to 16.5 to 23.5 dB and the front-to-back ratio exceeded 18 dB in the whole band. These properties met the requirements for high gain characteristics in SW broadcast antennas, ensuring the ability to transmit SW signals over thousands or even tens of thousands of kilometers.

**Figure 11:**
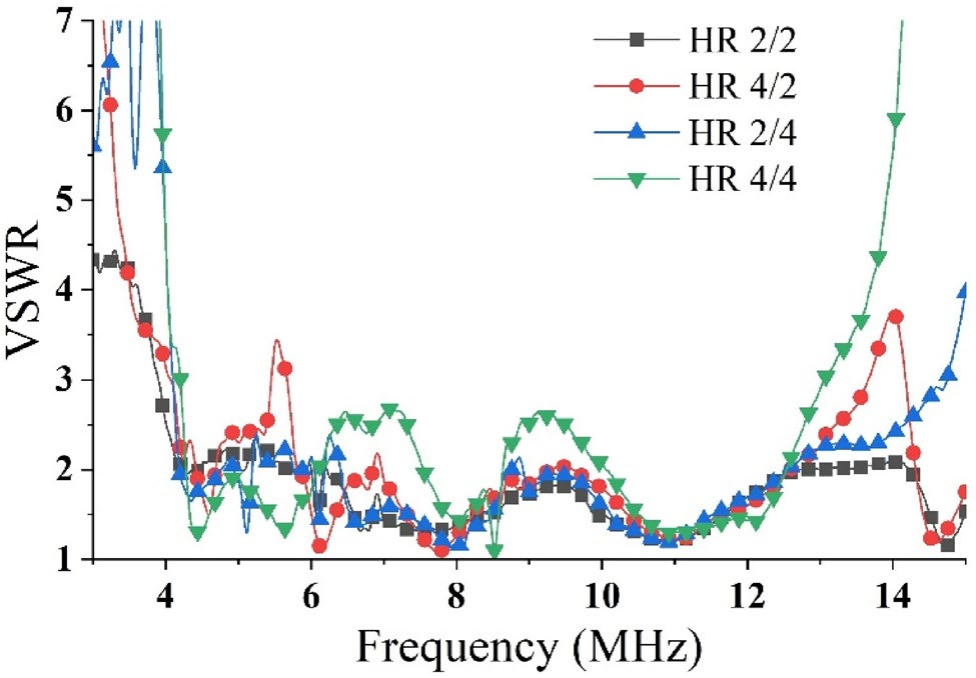
Feeding network participation, the VSWR of the total port in each mode.

With the participation of the feeder network shown in Fig. 8, the VSWR of the balanced port main feeder (characteristic impedance of 100 Ω) in each mode is shown in Fig. 11. In the target frequency band, the VSWR is less than 2.5, which is comparable to the level of each element in Fig. 9. The comparison between the efficiency of the array itself and the efficiency via the feeding network is also listed in Fig. 9. Obviously, the feeding network reduces the efficiency of some frequency points, but the overall efficiency remains above 70%, and some frequency points are higher than 95%.

The secondary array was a 1:2 scaled prototype of the primary array, which assumed the task of transmitting frequencies in the 13 to 27 MHz range. The VSWR of each mode of the higher-frequency array was lower than 2.5 in this frequency band. Owing to the half scaling, the radiation characteristics of the array in the 13 to 27 MHz range were similar to those of the initial array in the 6.5 to 13.5 MHz range. Table 2 lists the comparison of several large SW transmit antennas with this work. Including early fixed, non-rotatable antennas that required three arrays to barely cover the half SW band. And today's mainstream rotatable antennas: only two arrays can cover almost the entire SW bands. From these cases, it can be concluded that the proposed antenna achieves better performance (wider frequency band, more complete functions) in the smallest size. At the same time, the frequency of short-wave broadcasting is expanded to 4 MHz (the lowest frequency of the mainstream is still 6 MHz), which provides a possible approach to enhance the coverage of the near-range blind area.

**Table 2:** Comparison between existing SW broadcast arrays and proposed array.

Ref	Frequency band (MHz)	Antenna type	Number of units	Dimensions (λ_0_)	Gain (dBi)	Multi-mode
9	6–7 9–11 11–15	Cage antenna	2 × 4 2 × 8 2 × 6	2.78 × 2.8 3.87 × 5.4 2.13 × 3.78	18–20.8 19.4–21.2	Not available
10	12–27	Folded dipole	4 × 4	Not mentioned	Nearly 20	Not available
30	6–11 13–26.1	Folded dipole	4 × 4	4.07 × 4.17 × 0.83	14–23	Four modes
This work	4.4–27.4	Folded dipole	4 × 4	3.97 × 4.13 × 0.72	12–23.5	Four modes

### Fabrication and measurement of the scaled prototype

**Figure 12:**
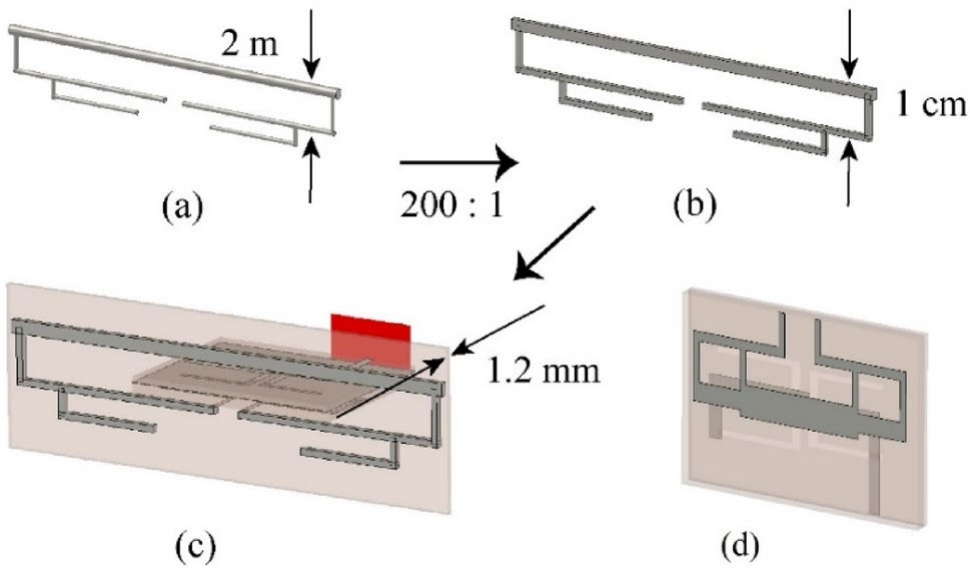
Scaled model of folded dipole: (**a**) initial dipole, (**b**) scaled down by 1:200, (**c**) fabricated on the PCB, (**d**) wideband PCB balun.

To verify its feasibility, the antenna array was tested using a scale model with a scale factor of 1:200. After being scaled down by this ratio, the frequency band lied in the microwave range (880–2700 MHz), which was suitable for measurement in the current mainstream microwave anechoic chamber. The scaled dipoles had thin lines and narrow gaps, and the model was manufactured using a printed circuit board, as shown in Fig. 12, to ensure the accuracy of the scale model. After shrinking from (a) to (b) in the figure, the folded dipoles exhibited identical impedance characteristics in their respective frequency bands. The balanced transmission lines became extremely thin (0.05 mm) after being scaled down, which were difficult to manufacture and install. The feed network was redesigned in the form of a microstrip.

To apply the unbalanced transmission method comprising a microstrip line and coaxial cable, each dipole needed to be connected to a balun. The impedance of the balanced port of the dipole was 600 Ω; therefore, the balun also needed to function as an impedance converter. The microstrip balun for the scaled dipole, shown in Fig. 12d, consisted of a balanced port, a slot layer, and an unbalanced port. The substrate used was FR4 with a relative permittivity of 4.3. The folded dipole was printed on both sides of the 1.2 mm thick FR4 substrate and connected via with a diameter of 0.4 mm to ensure its integrity. The various parameters of the scaled array (the spacing of each element, reflector size, and distance from the ground) were all reduced by 200 times according to the original design. Figure 10 shows the details of the scale model, where the unbalanced port of the balun was connected to the 50 Ω SMA interface by a coaxial cable. It should be emphasized that, owing to the original array being disposed on land (− y direction was an infinite conductive boundary), an actual experiment could not be conducted. Therefore, a finite metal surface was used to simulate the ground effect. Considering the feasibility of the actual test, the size of the ground metal plate was set to 1.2 × 1 m^2^.

**Figure 13:**
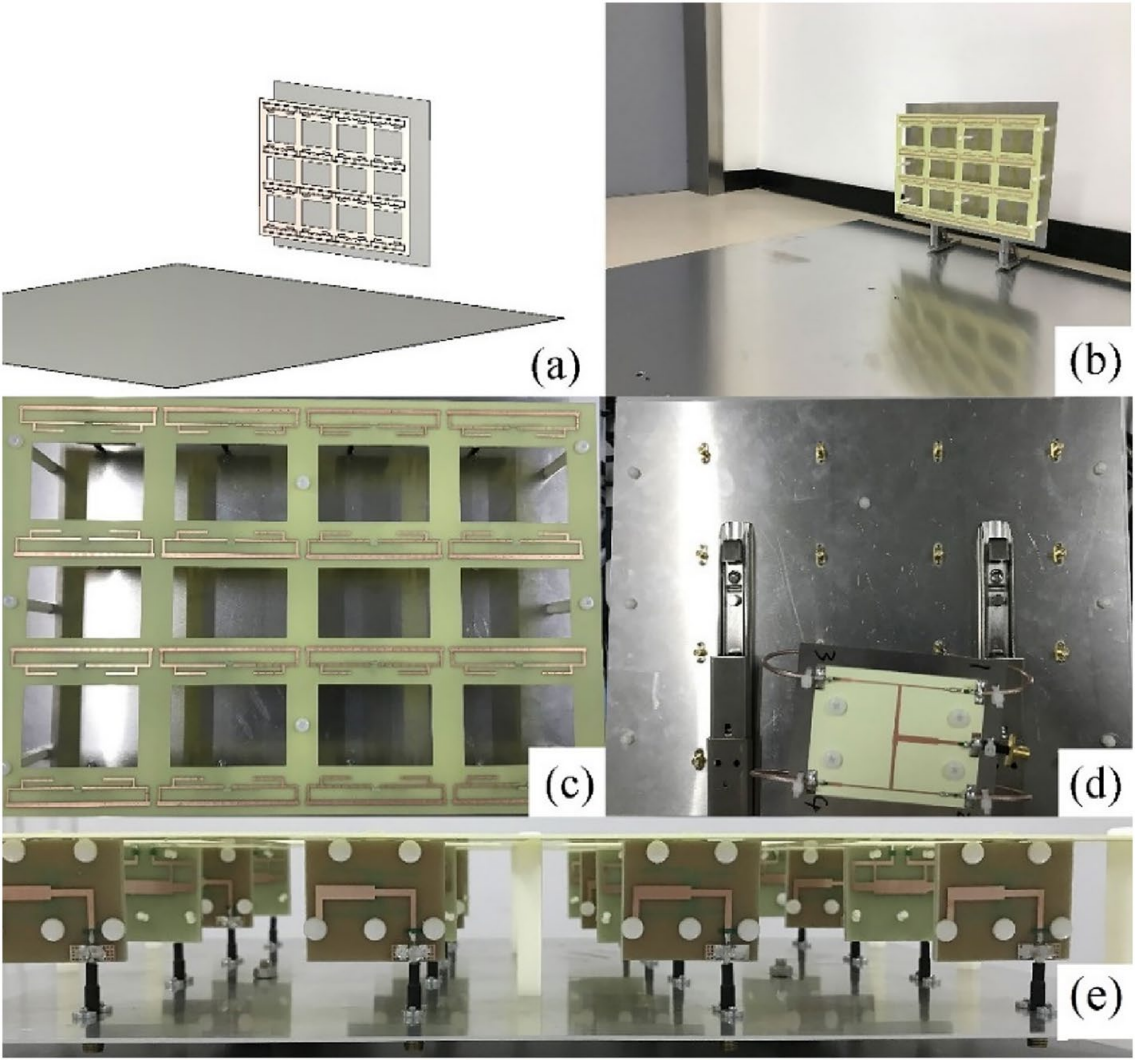
Scale model of the transmitting antenna array. (**a**) Simulation model, (**b**) perspective view of the fabricated model, (**c**) front view, (**d**) back view, and (**e**) top view.

**Figure 14:**
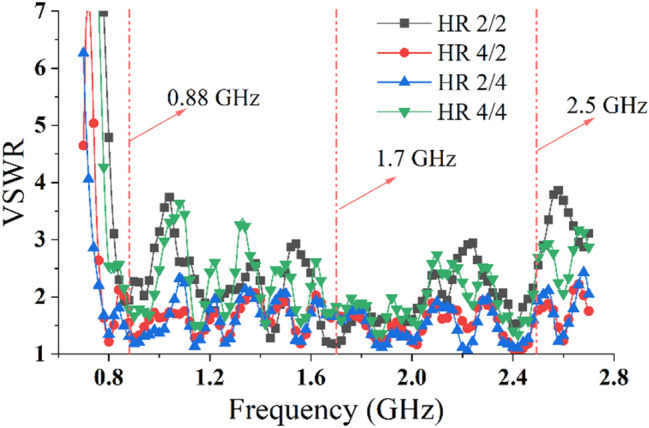
Measured VSWRs of the four modes.

The overall simulation model and fabrication sample of the 1:200 model are shown in Fig. 13a,b, respectively. Figure 13c shows the antenna array fabricated by a Printed Circuit Board (PCB) process, and the substrate is FR4. To achieve the various operating modes of the transmitting array, this scheme adopted a power divider to feed the antenna array. Figure 13d shows the power divider feeding of the HR 2/2 mode. Each array mode can be implemented using a cascade of multiple power dividers. Figure 14 shows the measured VSWRs of each mode using an Agilent Network Analyzer E5070B. From the measurement, it was evident that the starting frequency of the array was lower than that of a single dipole. This also verified that the mutual coupling can miniaturized the antenna array and achieved a wider band.

**Figure 15:**
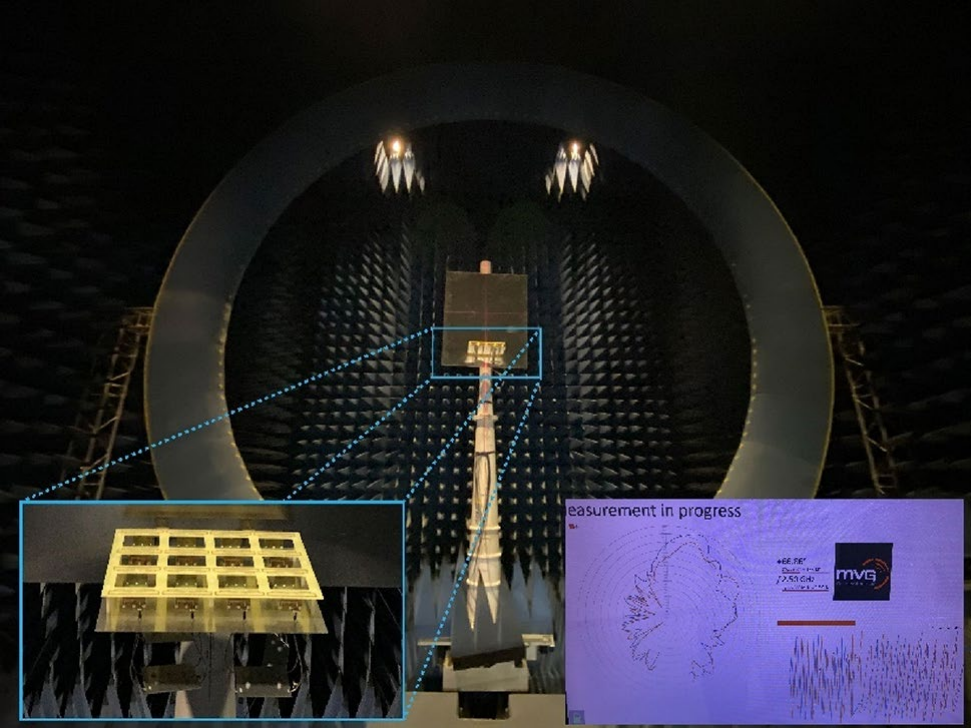
Multiprobe near-field measurement system.

This section details the radiation characteristics of the antenna array at 4.4, 8.5, and 12.5 MHz. This experiment compared the patterns at 880, 1700, and 2500 MHz of the scaled-down model with the three frequencies of the original antenna. The scaled array, characterized by the Satimo SG128 multiprobe near-field measurement system, had a test temperature 15 °C and a relative humidity of 80% (as shown in Fig. 15). Figure 10 presents the comparison between four normalized patterns at each frequency point in each mode. The four patterns included the simulated results of original antenna array, the simulated results of the scale model with infinite ground, and the measured and simulated results of the scale model with finite ground. Generally, a good agreement was obtained between each pattern, and the sidelobe level and angle essentially coincided. Notably, owing to the limited size of the metal plate simulating the ground, the antenna used in the test produced a small amount of downward radiation (under the infinite conductive boundary, the antenna would not radiate downward). Meanwhile, the gap between the elements was filled with substrate, causing the horizontal beam width to widen. This is more evident at 880 MHz and these changes widened the beam of the array and reduced the gain.

## Discussion

### Enhancement of the antenna array

**Figure 16:**
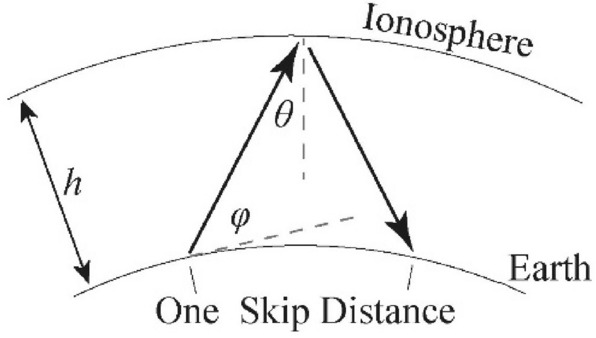
Sky wave propagation diagram.

The shortest distance traversed during sky wave propagation (i.e., one skip distance) is determined by the height of the ionosphere and the critical incident angle. Figure 16 shows the one-skip propagation of the sky waves, where the launch elevation angle was set to *φ*, the incident angle to the ionosphere was *θ*, and the ionosphere height was *h*. Furthermore, the critical angle for the total internal reflection (TIR) of the ionosphere was related to the transmission frequency. Under the premise of a certain incident angle *θ*, the maximum usable frequency (MUF) can be expressed as follows^32^:

2 $$ MUF = \frac{CF}{{\cos \theta }} $$

The critical frequency (CF) is also the highest frequency that can be reflected when radio waves are incident perpendicularly. When the emission frequency exceeded the MUF, radio waves escaped the ionosphere. Moreover, the reduction in frequency also reduced the incident angle of TIR, e.g., when the CF was taken to be 4 MHz and the MUF was taken to be 5.9 MHz, the minimum incident angle *θ* was calculated to be 47° (the launch elevation angle *φ* was 43°). The F-region ionosphere, which reflects shortwaves, is located at an altitude of 300 km above sea level; therefore, the shortest skip distance for 5.9 MHz radio waves is approximately 650 km. Reducing the MUF to 4.4 MHz resulted in the minimum incident angle θ also dropping to 27° (the launch elevation angle *φ* was 63°), and the shortest skip distance was reduced to 305 km accordingly.

**Figure 17:**
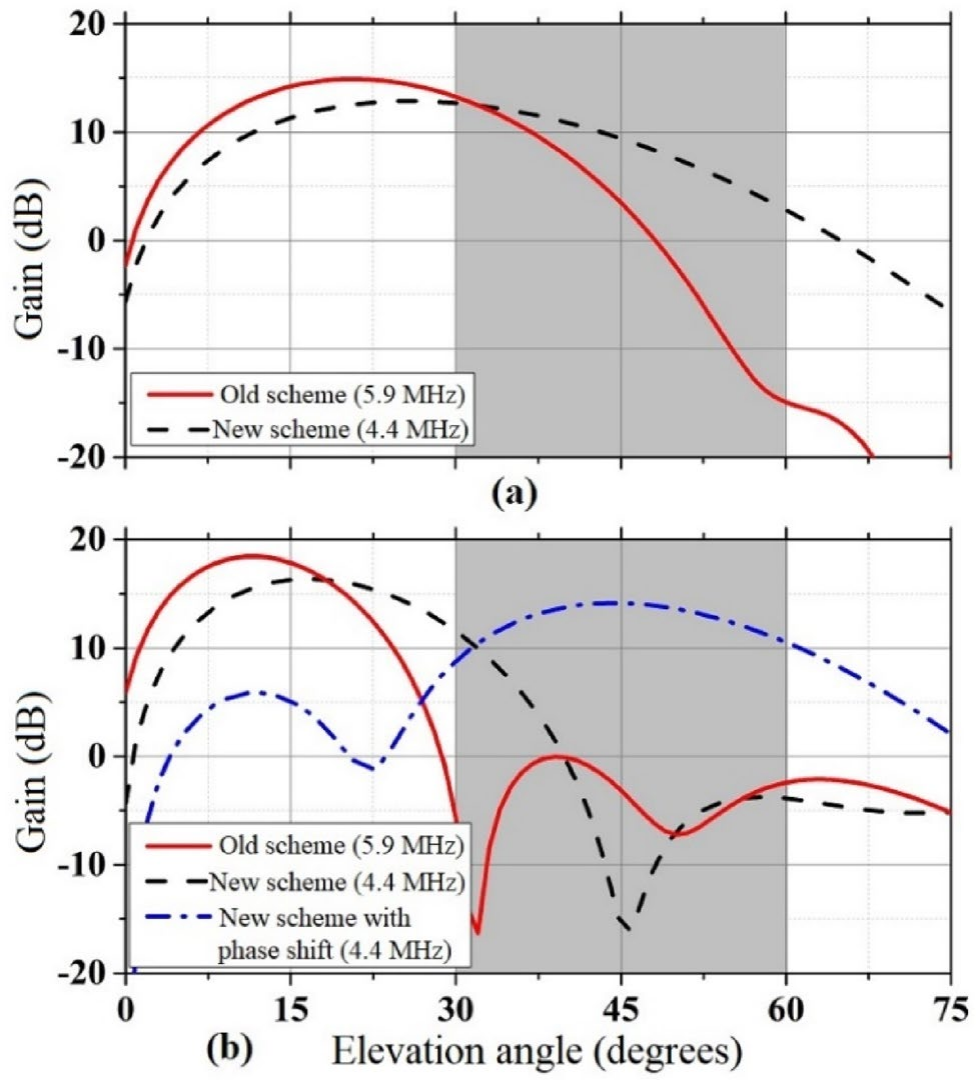
Comparison between the radiation patterns in the vertical plane for the new and old schemes: (**a**) HR 2/2 mode and (**b**) HR 4/4 mode.

The blind spot needed to be filled in the 400 to 1000 km range, and the corresponding elevation angle range was 30°–60°. For a decreased electron density of the ionosphere and a critical frequency below 4 MHz, the 5.9 MHz SW signal with an elevation angle over 43° would escape the ionosphere, whereas the 4.4 MHz SW signal could continue to propagate through the sky wave. In addition, the directional angle of the main beam would increase as the frequency decreased owing to reflection from the earth and the reflecting curtain, namely in the same feeding mode, a lower frequency implied a greater elevation angle. For an excitation of equal amplitude and phase, the comparison between the radiation patterns of antenna arrays at their lowest frequencies in the HR 2/2 and HR 4/4 modes are shown in Fig. 17. The target elevation in the 30°–60° range is shown in the figure. It was difficult to meet the needs of a specific angle with the pattern of the old scheme at the lowest frequency (as shown by the red line), especially owing to the weak gain of the HR 4/4 mode. The new scheme reduced the lowest frequency to 4.4 MHz, and the gain in the target angle range was found to be significantly improved. For the HR 2/2 mode, the larger the angle, the higher the gain. For the HR 4/4 mode, the new solution produced a larger gain in the 30°–40° range. Above 40°, the respective gains were negligible.

The elevation angle of the HR 4/4 mode remained poor; therefore, the phased array could be used to increase the angle of the main beam. Thereby, the high-gain mode could also be used for medium- and short-range target coverage. In the design, a 90° interelement phase shift was provided between the lines, and the elevation angle of the main lobe was significantly increased. Notably, within the target elevation, the proposed antenna array obtained a more superior gain than in the old scheme, and the gain value exceeded 10 dB (the value in the old scheme was below 0 dB). This advancement had improved the broadcast effect of the antenna near and mid-range transmission, making it suitable for a more comprehensive range of scenarios.

## Conclusions

SW broadcast antennas have currently presented new demands for covering the blind spots between traditional sky wave and surface wave propagation. In this study, a novel method was proposed for shortwave broadcast transmission achieved using a SW broadcast array in the 4.4–27.4 MHz range (the primary frequency array was in the 4.4–13.7 MHz range, and the secondary frequency array was in the 8.8–27.4 MHz range). In particular, the lower-frequency array had its bandwidth expanded by 36.6%, compared with that obtained from the common solutions (5.9–12.17 MHz) implemented at present, while retaining its original size. The wider operating band also benefitted from the mutual coupling between the elements. The impedance and radiation characteristics of the antenna array in each mode were simulated and described. Furthermore, a verification of the radiation properties of the proposed antenna showed that the gain gradually increased with the frequency from 12.8 to 23.5 dB. The front-to-back ratio and gain of the four specific modes all lied within the acceptable range, essentially meeting the requirements of SW broadcast transmission. Lastly, through a comparison with the results of a scaled prototype experiment, the simulation results were validated. The broadening of the antenna band to a lower frequency could effectively help the antenna array to increase the elevation angle of the radiation beam and reduce the critical angle of total reflection, thereby reducing the blind area.

## Data Availability

The data that support the findings of this study are available from the corresponding author upon reasonable request.

## References

[1] Wei YS, Tong P, Xu RQ, Yu L (2018). Experimental analysis of a HF hybrid sky-surface wave radar. IEEE Aerosp. Electron. Syst. Mag..

[2] Dzvonkovskaya A (2018). HF surface wave radar for tsunami alerting: from system concept and simulations to integration into early warning systems. IEEE Aero. Electronsp. Syst. Mag..

[3] Tian YW, Tian Z, Zhao JR, Wen BY, Huang WM (2020). Wave height field extraction from first-order doppler spectra of a dual-frequency wide-beam high-frequency surface wave radar. IEEE Trans. Geosci. Remote Sens..

[4] Wei Y, Peng T, Xu R, Lei Y (2018). Experimental analysis of a HF hybrid sky-surface wave radar. IEEE Aerosp. Electron. Syst. Mag..

[5] Yang LQ (2018). Simulation analysis and experimental study on the echo characteristics of high-frequency hybrid sky-surface wave propagation mode. IEEE Trans. Antennas Propag..

[6] Zhou QC, Gao HT, Zhang HJ, Wang F (2013). Robust superdirective beamforming for HF circular receive antenna arrays. Prog. Electromagn. Res..

[7] Roarty H (2019). The global high frequency radar network. Front. Mar. Sci..

[8] Fallis H, Folkert MB (1988). Practical techniques for feeding two or more high power shortwave transmitters to one antenna. IEEE Trans. Broadcast..

[9] Kershner SW (1968). Curtain antennas for high-power HF broadcasting applications. IEEE Trans. Broadcast..

[10] Martin JM, Guilbault F (1988). A new design for high performance HF curtain antennas. IEEE Trans. Broadcast..

[11] Wilensky R (1988). High-power, broad-bandwidth HF dipole curtain array with extensive vertical and azimuthal beam control. IEEE Trans. Broadcast..

[12] Beeke KL, Manton RG (1989). Vertical radiation-patterns of HF curtain arrays. Electron. Commun. Eng..

[13] Ali A, Ozbey B, Topcu S, Altintas A (2016). Feasibility study of installation of solar panels on a high-power HF antenna land. Int. J. RF Microw. Comput.‐Aided Eng..

[14] MacWilliam, K. & Schonken, F. Design of an HF transmitter antenna for bistatic ionospheric soundings in Antarctica. In *2020 IEEE Radar Conference *(Florence, Italy, 2020). https://ieeexplore.ieee.org/document/9266445.

[15] Hawkins JD, Lok LB, Brennan PV, Nicholls KW (2020). HF wire-mesh dipole antennas for broadband ice-penetrating radar. IEEE Antennas Wireless Propag. Lett..

[16] Ursenbach, M. J. M. Rotating antenna with wire dipoles. U. S. Patent 5 270 725 (1993).

[17] Ben-Dov, O. Broadcast antenna system with high power aural/visual self-diplexing capability. U. S. Patent 4 590 479 (1986).

[18] Menzel W, Pilz D, Al-Tikriti M (2002). Millimeter-wave folded reflector antennas with high gain, low loss, and low profile. IEEE Antennas Propag. Mag..

[19] Riddolls, R. J. A Canadian perspective on high-frequency over-the-horizon radar. *Defence R & D Canada* 379–384 (2007).

[20] Thayaparan T, Dupont D, Ibrahim Y, Riddolls R (2019). High-frequency ionospheric monitoring system for over-the-horizon radar in Canada. IEEE Trans. Geosci. Remote Sens..

[21] Orga F, Hervas M, Alsina-Pages RM (2016). Flexible low-cost SDR platform for HF communications near vertical incidence skywave preliminary results. IEEE Antennas Propag. Mag..

[22] Marcus C (2016). Walden, High-frequency near vertical incidence skywave propagation findings associated with the 5 MHz experiment. IEEE Antennas Propag. Mag..

[23] Ignatenko M, Filipovic DS (2016). On the design of vehicular electrically small antennas for NVIS communications. IEEE Trans. Antennas Propag..

[24] Kai R, Ranjbar NM, Nader B (2020). Design of dual-polarized, platform-based HF antennas using the characteristic mode theory. IEEE Trans. Antennas Propag..

[25] Witvliet BA, Maanen E, Petersen GJ, Westenberg AJ, Bentum MJ, Slump CH, Schiphorst R (2015). Near vertical incidence skywave propagation: elevation angles and optimum antenna height for horizontal dipole antennas. IEEE Antenn. Propag. Mag..

[26] Volakis JL (2018). Antenna Engineering Handbook.

[27] Phelan HR (1970). A wide-band parallel-connected balun. IEEE Trans. Microw. Theory Techn..

[28] Poisson A (1980). Conductivity-salinity-temperature relationship of diluted and concentrated standard seawater. IEEE J. Oceanic Eng..

[29] Bradshaw AL, Schleicher KE (1980). Electrical-conductivity of seawater. IEEE J. Oceanic Eng..

[30] Bouko, J., Aubry C. & Salvat, F. High-power rotating joint for a double-waveband antenna. German Patent DE 3 375 543, (1988).

[31] Tao JQ, Li GH, Mao J (2003). Rotating antenna radiating system for high-power SW broadcasting. Radio TV Broadcast Eng..

[32] Hubisz, John LJ (2014). Physics and chemistry of the upper atmosphere. Phys. Teach..

